# Description of two new species of *Clivina* Latreille (Coleoptera, Carabidae, Clivinini) from southeastern United States

**DOI:** 10.3897/zookeys.178.2817

**Published:** 2012-03-29

**Authors:** Yves Bousquet, Paul E. Skelley

**Affiliations:** 1 Agriculture and Agri-Food Canada, Central Experimental Farm, Ottawa, Ontario K1A 0C6, Canada; 2 Florida State Collection of Arthropods, Florida Department of Agriculture and Consumer Services, Gainesville, Florida 32614-7100, U.S.A.

**Keywords:** Florida, Alabama, taxonomy, *Clivina*

## Abstract

Two new species of the genus *Clivina* Latreille are described. One, *Clivina choatei* Bousquet & Skelley, belongs to the nominotypical subgenus and is known from six specimens collected in northern Florida. The species is structurally similar to *Clivina myops* Bousquet, known only from the holotype found in North Carolina, but differs among others by its smaller size and wider elytral striae. The second species, *Clivina alabama* Bousquet, belongs to the subgenus *Antroforceps* Barr and is known from two specimens collected in north-central Alabama. The species is structurally most similar to *Clivina sasajii* Ball, known only from Latimer County in Oklahoma, but differs among others in the absence of eyes and in having the pronotum and elytra proportionally wider.

## Introduction

The genus *Clivina* Latreille is a moderately diverse taxon represented in all continents, except Antarctica, by about 375 species. In the Nearctic Region, the genus contains 17 species of which three are adventive on this continent. These species are arrayed in five subgenera: *Paraclivina* Kult, *Clivina* s.str., *Semiclivina* Kult, *Antroforceps* Barr, and *Leucocara* Bousquet (= *Reichardtula* Whitehead *sensu auctorum* in part). Ball (2001) provided an excellent description of the adult structural characters of the genus and a key to the subgenera represented in North America.

The purpose of this paper is to describe two new species belonging to the nominotypical subgenus of *Clivina* from northern Florida and to the subgenus *Antroforceps* from north-central Alabama.

## Methods

The following measurements, made with an ocular micrometer in a Leica MZ16, were made on all specimens of the type series and from the holotype of *Clivina myops* and two paratypes of *Clivina sasajii*: head length (HL) – linear distance from apical edge of clypeus to posterior edge of left temple; length of pronotum (PL) – linear distance from anterior to posterior edge, measured along the midline; width of pronotum (PW) – greatest linear transverse distance; length of elytra (EL) – linear distance from parascutellar seta to apex, measured along the midline; width of elytra (EW) – greatest linear transverse distance across both elytra. The standardized body length (SBL) is the sum of the lengths of head, pronotum, and elytra, as specified above. The apparent body length (ABL) is the length measured from the apex of the mandibles to the apex of the abdomen.

## Taxonomy

### Clivina (Clivina) choatei

Bousquet & Skelley sp. n.

urn:lsid:zoobank.org:act:00E3504B-3898-4FF5-81C1-83097A64D4E4

http://species-id.net/wiki/Clivina_choatei

Fig. 1

#### Type material.

 Holotype, male, labeled: “FLORIDA: Levy Co. 4.0mi SW Archer 20-IX-30-XII-2001 P. Skelley, panel BIT / Holotype Clivina choatei Bousquet & Skelley.” The holotype is in the Florida State Collection of Arthropods, Gainesville, Florida (FSCA). Paratypes (five specimens): one specimen labeled “FLORIDA: Levy Co. 4.0mi SW Archer on Rt.24; 1-16-V-2001 P. Skelley, panel BIT”; 2 specimens labeled “FLORIDA: Levy Co. 4.0mi SW Archer 25-I-1992; Heyer, Skillman, Skelley Geomys chambers”; 2 females labeled “Gilchrist Co. N. Bell, 1mi.S.Rt.340 on Rt.129/40; 4-XII-1997 to 20-III-1998; P. Skelley *Geomys* burrow pitfall.” All paratypes are also labeled “Paratype Clivina choatei Bousquet & Skelley” and are deposited in FSCA (4 specimens) and in the Canadian National Collection of Insects, Ottawa, Ontario (1 specimen).

#### Etymology.

The specific epithet is a Latinized singular noun in apposition, genitive case, based on the surname of Paul “Skip” Choate (Department of Entomology & Nematology, University of Florida, Gainesville), who first collected this species and showed the specimens to PES. Unfortunately, his original specimens were lost.

#### Description.

*Color*. Body rufous with median and posterior legs slightly paler. *Microsculpture*. Clypeus and frons without meshes. Pronotum without meshes except at extreme base. Elytra without meshes except along intervals 8. Visible abdominal sternites 2–5 without meshes between ambulatory setae; visible sternite 6 with band of faint isodiametric meshes between ambulatory setae; visible sternites 1–2 with coarse, more or less isodiametric meshes laterally. *Head*. Labrum with six or seven dorsal setae. Clypeus with anterior margin almost straight, with very small lateral dentiform projections. Frontoclypeal suture indistinct. Frons without median fovea. Anterior supraorbital seta near level of posterior edge of eye. Eye small, flat. Antennomere 2 subequal in length to antennomere 3; antennomere 2 with several setae, most located in apical half; antennomeres 6–10 subquadrate. Mentum tooth proportionally large, not acuminate, apex more or less rounded, not quite reaching apex of lobe. *Thorax*. Pronotum with disc convex, with few faint wrinkles, without conspicuous punctures; lateral edges subparallel between level of setigerous punctures, with single denticle in posterior third; anterior angles protruding; median longitudinal impression shallow but relatively wide; anterotransverse impression distinct except near median impression, deep. Proepisternum with punctures on anterior half. Metepisternum with punctures. Metasternum smooth except for a few punctures laterally. *Elytra*. Striae wide, as wide or almost so as corresponding intervals, sides of striae wavy. Intervals flat; intervals 3 and 4 with conspicuous protuberance each at extreme base; interval 3 with five setigerous punctures; intervals 6 and 7 not carinate near base. Lateral edge along humeral region smooth, without indentations. *Abdomen*. Visible abdominal sternite 2 without coxal lines; visible sternites with coarse, shallow but dense punctures laterally. *Legs*. Protarsomere 1 with minute process on lateral side. *Male genitalia*. Median lobe with apex spatulate; endophallus without spines.

SBL = 4.45–5.05 mm.

**Figure 1.**
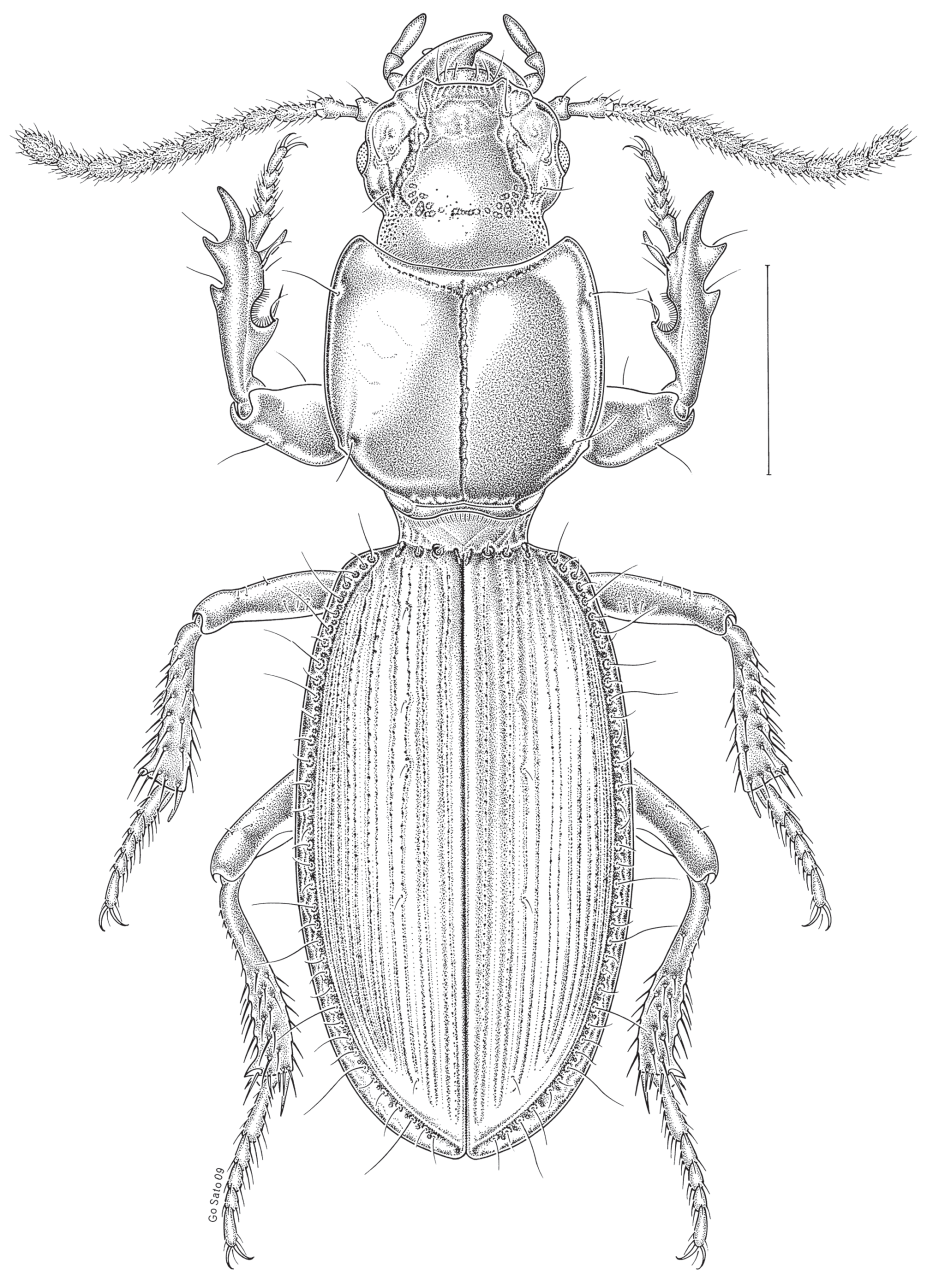
*Clivina choatei*, habitus (dorsal view); scale bar = 1 mm.

#### Habitat.

Although some specimens were collected in pitfall traps set in pocket gopher (*Geomys*) burrows, others were not. Choate’s missing specimens from western Alachua County were collected while excavating pocket gopher burrows, but may not have been in the burrows. The holotype and paratypes collected in “panel BIT” were far away from any rodent burrows. “BIT” stands for “burrow intercept trap” and was one of many passive underground trap designs the second author experimented with to collect subterranean insects (see Bousquet and Skelley 2010). Underground pitfalls sampling pocket gopher burrows sometimes act like a BIT and can collect other subterranean insects. Specimens of *Clivina choatei* and *Clivina sasajii* were collected in association with pocket gophers. This loose association is felt to be opportunistic for the beetle and a sampling bias by collectors looking for other underground insects.

All specimens of *Clivina choatei* were collected in deep sandy soils of Levy and Gilchrist counties which are part of the Northern Brooksville Ridge. This is one of many isolated sand systems in the southeastern US, which are known for having many endemic plants and animals. A brief discussion of this area is given in Bousquet and Skelley (2010).

#### Geographical distribution.

This species is currently known only from Levy and Gilchrist Counties on the Florida Panhandle.

#### Remarks.

This species is structurally very similar to *Clivina myops*, which is known only from the holotype collected at Raleigh in North Carolina. The holotype of *Clivina myops* differs from specimens of *Clivina choatei* by its larger size (SBL = 6.40 mm), in having the median longitudinal impression of the pronotum deeper, the elytral striae proportionally narrower, their width being clearly less than that of the corresponding intervals, and the visible abdominal sternites 1 and 2 less punctate, with only a few, relatively small punctures. The ratios of various body proportions (Table 1) suggest that the pronotum is proportionally wider in *Clivina choatei* than in *Clivina myops*. The shape of the median lobe of the aedeagus did not differ significantly between the two species.

**Table 1.** Ratios for *Clivina* studied.

species	N	HW/PW	PW/EW	PL/PW	PL/EL	EL/EW
*Clivina choatei*	6	0.72–0.80	0.82–0.90	0.92–0.95	0.39–0.41	1.90–2.05
*Clivina myops*	1	0.79	0.77	0.98	0.39	1.92
*Clivina alabama*	2	0.70–0.78	0.86	0.96–0.98	0.45–0.47	1.79–1.84
*Clivina sasajii*	2	0.73–0.75	0.87–0.92	1.11–1.17	0.47–0.48	2.13–2.17

In Bousquet’s (1997) key to the Nearctic species of the subgenus *Clivina*, *Clivina choatei* will key out to couplet 6. The following modification should be made to incorporate the new species:

**Table:** 

6	Antennomere 2 at most with one seta on apical half. Proepisternum smooth or at most with a few punctures	*Clivina pallida* Say
–	Antennomere 2 with several setae on apical half. Proepisternum punctate on anterior half	6A
6A	Elytral striae proportionally narrower, width smaller than that of corresponding intervals. Body larger, ABL = 7.0 mm	*Clivina myops* Bousquet
–	Elytral striae proportionally wider, width subequal to that of corresponding intervals. Body smaller, ABL = 4.8–5.5 mm	*Clivina choatei* sp. n.


### Clivina (Antroforceps) alabama

Bousquet sp. n.

urn:lsid:zoobank.org:act:4937B489-B81C-496F-A78F-5925D330D7F6

http://species-id.net/wiki/Clivina_alabama

Fig. 2

#### Type material.

Holotype labeled: “AL: 0.5 mi S Highland Lake Blount Co. Oct. 22, 2009 T.N. King / Blind Carabid HL - 10.22.09 Rock 1000’ [handwritten] / Holotype Clivina alabama Bousquet CNC No. 24034.” The holotype is in the Canadian National Collection of Insects, Ottawa, Ontario. Paratype (1 specimen) labeled: “AL: 0.5 mi S Highland Lake Blount Co. April 2009 T.N. King / Paratype Clivina alabama Bousquet.” The specimen is deposited in R. Michael Brattain Collection (Lafayette, Indiana).

#### Etymology.

The specific epithet is a Latinized singular noun in apposition, nominative case, based on the name of the Alabama, a native American tribe who lived in southeastern United States.

#### Description.

With the character states of the subgenus *Antroforceps* as described by Ball (2001: 138–144) and the followings: *Color*. Body uniformly rufous, with antennae, palpi and most of legs paler. *Microsculpture*. Frons and clypeus without microsculpture. Pronotum without microsculpture except for small area near the posteriolateral dentiform projections. Elytra without microsculpture. Proepisternum with coarse, isodiametric meshes, sculpticells convex. Visible abdominal sternites 1–6 with coarse isodiametric meshes except medially on sternites 2–4, sculpticells convex. *Head*. Clypeus with anterior margin slightly concave medially, without dentiform projections laterally. Frontocly- peal suture very shallow. Frons without median fovea. Eye absent. Antennomeres 6–10 submoniliform. Mentum tooth proportionally small, apex more or less rounded, not reaching apex of lobe. *Thorax*. Pronotum slightly transverse (see Table 1); lateral edges shallowly crenulate; anterior angles acutely protruding; median longitudinal impression narrow along posterior half, widened along anterior half; posteriolateral dentiform projections blunt, basal projection as large as apical projection. *Elytra*. Lateral edges minutely crenulate on basal fourth; interval 4 convex in basal fourth; interval 5 rather flat; interval 6 and 7 carinate through most of length; interval 3 with five setigerous punctures.

SBL = 3.63–3.69 mm.

**Figure 2.**
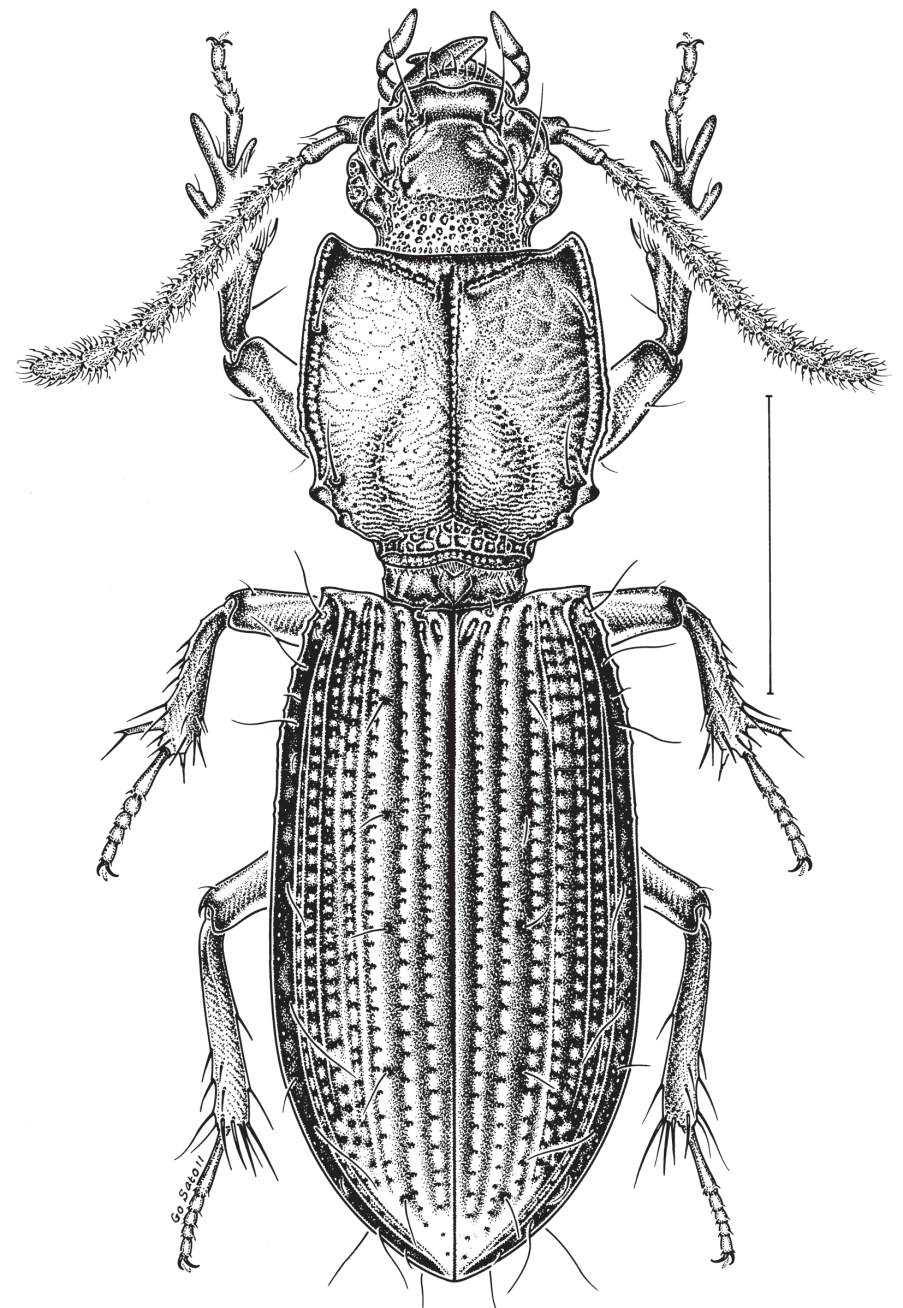
*Clivina alabama*, habitus (dorsal view); scale bar = 1 mm.

#### Habitat.

The two specimens were collected under rocks on a hillside in a mixed Pine/hardwood area above a stream following heavy rains (R. Michael Brattain, personal communication 2011).

#### Geographical distribution.

This species is currently known only from Blount County in north-central Alabama.

#### Remarks.

This species is structurally most similar to *Clivina sasajii*, which is known from nine specimens collected in Latimer County, eastern Oklahoma. The two paratypes of *Clivina sasajii* studied differ from those of *Clivina alabama* in having small eyes, the pronotum clearly elongate (see Table 1) and the elytra proportionally narrower (see Table 1), the anterior angles of the pronotum less projected, the posteriolateral dentiform projections more acute, with the basal projection smaller than the apical one, the microsculpture near the posteriolateral dentiform projections more expanded, the lateral edges of the elytra indistinctly crenulate on basal fourth, the interval 4 not convex, and the interval 6 carinate only on anterior fourth. The genitalia of the two specimens of *Clivina alabama* have not been extracted to preserve their integrity.

In Ball’s (2001: 144–145) key to the species of subgenus *Antroforceps*, *Clivina alabama* will key out to couplet 2. The following modification should be made to incorporate the new species:

**Table:** 

2	Elytral interval 3 with eight discal setae. Lateral margins of pronotum pronouncedly crenulate. Pronotum and elytra with paramedian dorsal projections	*Clivina bolivari* Barr
–	Elytral interval 3 with five discal setae. Lateral margins of pronotum shallowly crenulate. Pronotum and elytra without paramedian dorsal projections	3
3	Eyes absent. Pronotum slightly transverse (PL/PW = 0.96–0.98); elytra proportionally wider (EL/EW = 1.79–1.84)	*Clivina alabama* sp. n.
–	Eyes present, small. Pronotum elongate (PL/PW = 1.11-1.17); elytra proportionally narrower (EL/EW = 2.13–2.17)	*Clivina sasajii* Ball

## Supplementary Material

XML Treatment for Clivina (Clivina) choatei

XML Treatment for Clivina (Antroforceps) alabama

## References

[B1] Ball GE (2001) The subgenera of *Clivina* Latreille in the Western Hemisphere, and a revision of subgenus *Antroforceps* Barr (new status), with notes about evolutionary aspects (Coleoptera: Carabidae: Clivinini). Special Publication of the Japan Coleopterological Society of Osaka 1: 129-156

[B2] Bousquet Y (1997) Description of a new species of *Clivina* Latreille from southeastern United States with a key to North American species of the *fossor* group (Coleoptera: Carabidae: Clivinini). The Coleopterists Bulletin 51: 343-349

[B3] Bousquet Y Skelley PE (2010) Description of a new species of *Scarites* Fabricius (Coleoptera: Carabidae) from Florida. The Coleopterists Bulletin 64: 45-49 doi: 10.1649/0010-065X-64.1.45

